# CORRELATION BETWEEN VISUAL GAIT ANALYSIS AND FUNCTIONAL ASPECTS IN CEREBRAL PALSY

**DOI:** 10.1590/1413-785220162405162986

**Published:** 2016

**Authors:** MAIRA RECH FOLLE, ANA PAULA TEDESCO, RENATA D´AGOSTINI NICOLINI-PANISSON

**Affiliations:** 1. Instituto de Neuro-Ortopedia, Caxias do Sul, RS, Brazil.

**Keywords:** Gait, Cerebral palsy, Evaluation.

## Abstract

**Objective:**

To verify the correlation between visual gait analysis (VGA) by the Edinburgh visual gait score (EVGS) and functional aspects using the Timed Up and Go Test (TUG) and Gross Motor Function Classification System (GMFCS) in individuals with cerebral palsy (CP).

**Methods:**

Retrospective cross sectional study of 35 patients with CP. The mean age 12.61 years old, 94.3% were spastic; 34.4% hemiplegic, 54.3% diplegic, 11.4% triplegic; 45.7% were level II GMFCS, 42.9% level I, 5.7% level III and 5.7% level IV. VGA was analyzed by the Edinburgh visual gait score (EVGS), functional mobility was assessed by TUG and functionality through GMFCS. The Spearman correlation was used for statistical analysis.

**Results:**

The mean EVGS score was 18.97. The mean TUG was 13.71sec. EVGS showed moderate correlation with TUG (r=0.46, p=0.03) and GMFCS (r=0.45, p=0.00).

**Conclusion:**

Worse VGA scores correlate to worse functionality and mobility performance. Due to the observed correlation, it is possible to assert that VGA is a useful tool on evaluation of CP patients. Level of Evidence III, Retrospective Comparative Study.

## INTRODUCTION

The gait analysis is an important tool for assessing changes in gait of neurological patients. The gold standard for evaluation is computadorized gait analysis (CGA).

The importance of gait assessment in cerebral palsy (CP) has been found in a study that showed that CGA has a strong impact on surgical treatment decision and that worst results were recorded by surgeons who did not follow what was proposed by CGA.^1^

CGA, however, is an expensive and unavailable technology in many centers, so that visual gait analysis (VGA) with video recording may assist the process of study, diagnosis and treatment indication.^2^ VGA is indicated for normal and pathological gait assessment, whenever CGA is not available; in very young children, under 4 to 6 years old; and in individuals with little comprehension and cooperation.^1^ ^-^ ^3^

One of the disadvantages that have been described regarding VGA is the difficulty in visualizing the transverse plane. However, the placement of rotation indicators (markers) in the pelvis and thigh may aid visualization of pelvis and hip rotations.^4^

The literature shows that even CGA, which provides accurate and objective parameters, relies on subjective data interpretation and is, therefore, variable, especially if they are analyzed in different laboratories, also depending on the examiners' experience and reliability of clinical examination and data collection techniques.^5^ ^-^ ^7^ CGA can still show differences in the results due to errors of the own computer system and software, markers placement technique, or slight performance variability of individuals between tests.^8^

Thus, even if VGA is unable to discriminate the quality of movement as CGA, it is considered a useful tool of moderate reliability, which is greatly influenced by the clinical experience of the observer.^2^ ^,^ ^9^ ^-^ ^11^

There are several protocols described in the literature to evaluate the VGA in CP, such as the Physician's Rating Scale (PRS),^12^ the Edinburgh Visual Analysis Score (EVGS)^13^ and the Observational Gait Scale,^14^ among others.

EVGS was developed as a VGA score for patients with CP. Gait videos in coronal and sagittal planes are analyzed according to 17 parameters for each inferior limb, which correspond to key elements of normal and pathological gait, graduates in a three scores range (0: normal, 1: moderate and 2: marked), the maximum score being 34. Six different anatomical levels are analyzed: trunk, pelvis, hip, knee, ankle and foot, in the transverse, coronal and sagittal planes and support and balance phases of the gait.^13^ EVGS had its inter and intraobserver reliability and sensitivities evaluated in studies that compared it with CGA, with a correlation around 64% of the items assessed.^13^ It has also been compared to other measurements, such as the Gillette Gait Index, Gillette Functional Assessment Questionnaire and speed, and showed significant correlations, especially with Gillette Gait Index.^15^

The intra and inter-observer reliability of the scale depends on the observers' experience and training - the higher the experience, the greater the intraobserver reliability.^16^ ^-^ ^20^ A study concluded that the observation of gait events by inexperienced observers using EVGS was moderately reliable, however, there was little precision when compared to experienced observers and to CGA.^19^ Another study comparing PRS and EVGS, showed excellent intraobserver reliability, but the inter-observer reliability for both scales was considered low. The study recommended that VGA was made by the same observer.^16^

The reliability and validity of EVGS and five other tools was compared with the CGA. EVGS was considered the best tool to assess the gait pattern in CP, because it considers motion data in the three planes, with good reliability and concurrent validity.^4^ ^,^ ^19^ ^-^ ^22^

There are studies that correlate data from VGA with functional mobility measurements and levels of functionality. There are a correlation between data from CGA and EVGS and the Gross Motor Function Classification System levels (GMFCS), demonstrated that high scores on both gait assessment methods matched with high levels of GMFCS.^21^ In another study, the Timed Up & Go method (TUG) was analyzed, along with other functional mobility elements and showed strong correlation with functional gait ability of individuals with CP.^23^ Kerr et al.^24^ have shown that low levels of GMFCS were associated to low values ​​of the Pediatric Evaluation of Disability Inventory.

GMFCS was developed to classify the functional abilities of children with CP. This scale is currently considered the most reliable and best known in pediatric CP rehabilitation.^25^

TUG is a quick and easy test that assesses the functional mobility and consists in measuring the time spent to go from the sitting position to orthostatic posture, start walking at the command, stop, return, come back, and sit down again. This test was validated and adapted for children and adolescents with CP^26^ and has also has normal values ​​for children and adolescents.^27^

With this perspective, the objective of this study was to verify the correlation between gait pattern measured by EVGS, functional mobility (TUG) and the level of functionality (GMFCS) of individuals with CP.

## MATERIALS AND METHODS

This is a cross-sectional retrospective review study of medical records of patients with CP who underwent VGA analyzed using EVGS between January 2010 and March 2015, as part of their evaluation at *Instituto de Neuro-Ortopedia*. The records with incomplete data were excluded. This study was approved by the Research Ethics Committee under number 1127171 (CAAE: 43305015.5.0000.5668).

In the VGA assessment routine, anatomical marks were made to facilitate observation of the videos. Besides marks made with white paint on the patella and posterior side of the calcaneus, as well as markers in the anterior-superior iliac crests, as described in the original study,^13^ markers in the sacrum and dorsal surface of the feet (at the level of the second metatarsal), respectively to better identify deviations in the transverse and coronal plane of the legs.^4^ For individuals showing difficulty to walk in a straight line, markers were placed on the floor with tape for the purpose of orientation. In this study, the data collected from VGA, performance in the TUG test and classification by GMFCS were analyzed, aiming to demonstrate correlation between them.

VGA was performed using videos in the coronal and sagittal planes with extra focus on the feet. The videos were analyzed using the criteria described in the EVGS and the total score was calculated by summing the score of both limbs.

The TUG test was conducted according to the methodology described by Williams.^26^ Three repetitions of the path were performed and the only the best time was considered. That is, the lower the number, the better the functional mobility.

GMFCS has five classification levels based on functional abilities and the CP child's movement initiative, emphasizing sitting and walking set by age groups. Distinctions between the levels are based on functional limitations, need for assistive technology, including the use of assistive devices (crutches, walkers and canes) and wheelchair.^25^

### Statistical analysis 

For statistical purposes, the normality of continuous variables was assessed by the Shapiro-Wilk test. Data with normal distribution were presented as mean and standard deviation and asymmetric data with median and interquartile range. Categorical variables were expressed as absolute and relative frequency. The association between the total Edinburgh score (sum of both lower limbs) with GMFCS and TUG was performed using Spearman's correlation test. All analysis and data processing were performed using SPSS version 18.0 (SPSS Inc., USA). In all cases, differences were considered significant when p<0.05.

## RESULTS

The review included data from 35 patients who underwent VGA and 28 subjects that were also evaluated through the TUG test. Table 1 shows the characteristics of the participants of the study. Both correlations of EVGS with TUG and GMFCS showed a moderate magnitude, according to Table 2. 

**Table 1:** Demographic and clinical characteristics of the sample of individuals with CP participating at the study.

Total population (n=35)	
Demographic characteristics	
Age (years old)	12.61±6.46
Male gender, n (%)	22 (62.9)
Clinical characteristics	
Injury topography, n (%)	
Hemiplegia	12 (34.3)
Diplegia	19 (54.3)
Triplegia	4 (11.4)
CP category, n (%)	
Spastic	33 (94.3)
Mixed	2 (5.7)
Classification, n (%)	
Unilateral	12 (34.3)
Bilateral	23 (65.7)
GMFCS, n (%)	
I	15 (42.9)
II	16 (45.7)
III	2 (5.7)
IV	2 (5.7)
TUG (n=28)	10 (8-15)
Edinburgh total	19 (9-26)
Right lower limb Left lower limb	8 (5-14) 11 (3-14)

Continuous data expressed as mean ± standard deviation or median (25-75 quartil). CP: Cerebral palsy; GMFCS: Gross Motor Function Classification System; TUG: Timed Up and Go test.

**Table 2:** Correlation of Edinburgh's Visual Gait Analysis with GMFCS and TUG.

	GMFCS	Magnitude	TUG	Magnitude
Edinburgh	0.45 (0.00)	Moderate	0.46 (0.03)	Moderate

Data expressed by the Spearman correlation coefficient r (p-value); GMFCS: Gross Motor Function Classification System; TUG: Timed Up and Go test.

## DISCUSSÃO

Correlations of EVGS with TUG and GMFCS, found in our study, were considered as moderate, showing the relationship between gait pattern, functional mobility and level of functionality. It has been shown that individuals with high scores on EVGS, i.e., major changes in gait pattern took more time to carry out the functional mobility test (TUG) and had worst level of functionality (GMFCS).

Similar results were obtained by Robinson et al., ^21^ who demonstrated a strong relationship between the results of CGA and EVGS, reinforcing the latter as an appropriate tool for examiners who do not have access to CGA. In this study, authors found a strong relationship between both CGA and EVGS with level of GMFCS I-III, and it has been observed that high gait scores were associated to high levels of GMFCS.^21^

A study that analyzed the gait performance through speed and the Gross Motor Function Measure (GMFM) demonstrated its relationship with the dimensions D (standing) and E (walking, running and jumping). The authors emphasized that the video recording enables better analysis of movements and assists the selection and evaluation of gait training strategies.^28^

The correlation between functional tests as TUG and the levels of GMFCS was demonstrated in a study that investigated the gait behavior in adult patients with CP. The authors reported decline in gait function, as compared with adolescents, and in 39% of cases there was change in the level of GMFCS. A correlation between TUG, GMFCS and Functional Mobility Scale with the six-minute walk test, evaluated in the study, showing that TUG had a direct influence on the functional gait ability.^23^

The literature reports GMFCS findings correlate with other mobility scales, such as the Pediatric Evaluation of Disability Inventory, for example, demonstrating that the lower the GMFCS level, the lower the mobility score at the Pediatric Evaluation of Disability Inventory.^24^ However, this study did not directly analyzed gait abnormalities, as in our study.

## CONCLUSION

It was possible to demonstrate that VGA, analyzed by the Edinburgh protocol, is able to correlate gait abnormalities with functional capacity, measured by the TUG test and GMFCS. Worse scores correlate with worse performance in terms of functionality and mobility in CP. EVGS seems to be an appropriate tool to evaluate the progress of patients with CP. 
